# The value of dual-energy spectral CT in differentiating solitary pulmonary tuberculosis and solitary lung adenocarcinoma

**DOI:** 10.3389/fonc.2022.1000028

**Published:** 2022-11-30

**Authors:** Guojin Zhang, Shenglin Li, Ke Yang, Lan Shang, Feng Zhang, Zixin Huang, Jialiang Ren, Zhuoli Zhang, Junlin Zhou, Hong Pu, Qiong Man, Weifang Kong

**Affiliations:** ^1^ Department of Radiology, Sichuan Provincial People’s Hospital, University of Electronic Science and Technology of China, Chengdu, China; ^2^ Department of Radiology, Chinese Academy of Sciences Sichuan Translational Medicine Research Hospital, Chengdu, China; ^3^ Department of Radiology, Lanzhou University Second Hospital, Lanzhou, China; ^4^ Department of Pharmaceuticals Diagnosis, GE Healthcare, Beijing, China; ^5^ Department of Radiology and BME, University of California Irvine, Irvine, CA, United States; ^6^ School of Pharmacy, Chengdu Medical College, Chengdu, China

**Keywords:** spectral CT, NSCLC, pulmonary tuberculosis, lung adenocarcinoma, differential diagnosis

## Abstract

**Background:**

To explore the value of dual-energy spectral CT in distinguishing solitary pulmonary tuberculosis (SP-TB) from solitary lung adenocarcinoma (S-LUAD).

**Methods:**

A total of 246 patients confirmed SP-TB (n = 86) or S-LUAD (n = 160) were retrospectively included. Spectral CT parameters include CT_40keV_ value, CT_70keV_ value, iodine concentration (IC), water concentration (WC), effective atomic number (Zeff), and spectral curve slope (λ_70keV_). Data were measured during the arterial phase (AP) and venous phase (VP). Chi-square test was used to compare categorical variables, Wilcoxon rank-sum test was used to compare continuous variables, and a two-sample *t*-test was used to compare spectral CT parameters. ROC curves were used to calculate diagnostic efficiency.

**Results:**

There were significant differences in spectral CT quantitative parameters (including CT_40keV_ value [all *P<* 0.001] , CT_70keV_ value [all *P<* 0.001], λ_70keV_ [*P<* 0.001, and *P* = 0.027], Zeff [*P =*0.015, and *P* = 0.001], and IC [*P =*0.002, and *P* = 0.028]) between the two groups during the AP and VP. However, WC (*P =* 0.930, and *P* = 0.823) was not statistically different between the two groups. The ROC curve analysis showed that the AUC in the AP and VP was 90.9% (95% CI, 0.873-0.945) and 83.4% (95% CI, 0.780-0.887), respectively. The highest diagnostic performance (AUC, 97.6%; 95% CI, 0.961-0.991) was achieved when all spectral CT parameters were combined with clinical variables.

**Conclusion:**

Dual-energy spectral CT has a significant value in distinguishing SP-TB from S-LUAD.

## Introduction

Lung cancer (LC) remains the leading cause of cancer-related death worldwide (1). Non-small cell lung cancer (NSCLC) accounts for more than 80% of LC cases, of which lung adenocarcinoma (LUAD) is the most common histological subtype (2). In clinical practice, some benign lesions have clinical symptoms and radiological findings that are similar to those of LC. Therefore, these benign lesions are often misdiagnosed as LC and receive overtreatment, such as surgical resection or needle biopsy (3, 4). Pulmonary tuberculosis (TB) is a radiographically diverse. When it presents as a solitary nodule or mass, it is difficult to distinguish from solitary LC, especially solitary lung adenocarcinoma (S-LUAD). Therefore, accurately distinguishing solitary pulmonary tuberculosis (SP-TB) and S-LUAD is a common and challenging issue.

Several imaging techniques, including CT (5), MRI (6), and PET/CT (7), have been used to differentiate between the two diseases. However, none of these methods yield satisfactory results. Currently, histological diagnosis remains the most accurate method to distinguishing between these two diseases. However, this method has some limitations. First, histological examination is an invasive procedure that may cause serious complications such as pneumothorax (8), hemorrhage (9), and air embolism (10). Second, needle biopsy increases the risk of TB dissemination (11). Finally, a small subset of tissues obtained from histological samples lacks overall information about the lesion (12). Thus, there is an urgent need for a non-invasive, accurate and convenient technique to differentiate between SP-TB and S-LUAD.

In recent years, with the widespread application of dual-energy spectral CT in clinical practice, new perspectives have been developed to study the internal characteristics of tumors, especially thoracic tumors. Dual-energy spectral CT uses instantaneous (<0.05ms) dual kVp (80 kVp and 140 kVp) switching technology to obtain single-energy images at different keV levels (40–140 keV). It can not only reflect the morphological characteristics of lesions but also provide multiparameter information such as single- spectral CT value, effective atomic number (Zeff), iodine concentration (IC), and water concentration (WC) of lesions. At the same time, it can also generate characteristic performance spectral curves and curve slopes for different lesions and tissues (4, 13). Studies have shown that spectral CT has achieved good performance in differentiating benign and malignant thoracic tumors (4), distinguishing histological subtypes of NSCLC (14), evaluating NSCLC pathological grades (15), and predicting the efficacy of chemotherapy for lung cancer (16). Therefore, the purpose of this study was to effectively distinguish SP-TB from S-LUAD by using dual-energy spectral CT.

## Materials and methods

### Patients

We retrospectively screened and collected spectral CT and clinical data of patients in Lanzhou University Second Hospital (Lanzhou, China) from January 2018 to March 2021. This retrospective study was approved by the Institutional Review Board, and the requirement for informed consent was waived. Inclusion criteria were as follows (1): histologically confirmed lung adenocarcinoma or pulmonary TB after surgery or biopsy (2); For TB patients, positive real-time fluorescence quantitative polymerase chain reaction test results (3); no relevant treatment before CT scan (4); presented as solitary nodules or masses on CT images (5); past dual-phase enhanced spectral CT scans; and (6) lesions without characteristic calcification or fat density on CT imaging. The exclusion criteria were as follows (1): history of other malignancies (2); time interval > 4 weeks between the CT scan and surgery or biopsy (3); lesions< 10 mm in maximum diameter (4); viral infections, such as HIV and HBV; and (5) age< 18 years. Smoking history was defined as non-smokers (never smoked) and smoking (previously or currently smoked). If the tumor crosses the fissure, the lobe location is defined as the lobe in which the tumor predominates.

According to the above criteria, 246 patients (105 females and 141 males; mean age ± standard deviation [SD], 53.96 ± 12.45 years; median age, 55.0 years) were enrolled, of which 86 were SP-TB (mean age ± SD, 46.98 ± 12.84 years; median age, 49.5 years) and 160 were S-LUAD (mean age ± SD, 57.71 ± 10.49 years; median age, 59.0 years).

### CT scanning protocol

All patients underwent dual-phase contrast-enhanced CT scans using the Discovery CT750 HD scanner (GE Healthcare, Milwaukee, WI, USA) in gemstone spectral imaging (GSI) mode. The CT scan parameters were as follows: tube voltage, 80 kVp and 140 kVp instantaneous (< 0.5ms) switching; tube current, 375 mA; beam pitch, 0.984:1; rotation time, 0.7 s; matrix, 512×512; scanning field, 50 cm; slice thickness, 5mm; and slice spacing, 5mm. Scanning range was from lung tip to lung bottom. The non-ionic contrast agent iohexol (300 mgI/ml, Yangtze River Pharmaceutical Group, Jiangsu, China) was injected through the median cubital vein using a high-pressure syringe (XD8000, Ulrich, Germany) at a dose of 1.3–1.5 mL/kg and an injection rate of 3.5–4.0 ml/s. Arterial phase (AP) and venous phase (VP) scans were performed 30 s and 60 s after contrast injection using automatic tracking technology. Moreover, 60% ASiR-V iteration was used to reconstruct images at the end of the scan with a reconstructed slice thickness of 1.25 mm and reconstructed slice spacing of 1.25 mm.

### Image analysis

Raw CT images were transferred to an ADW4.6 image post-processing workstation (GE Healthcare, Milwaukee, WI, USA). Two radiologists with 6 and 8 years of experience in thoracic diagnosis independently analyzed the images and reached a consensus by discussion in case of disagreement. Both physicians were blinded to the patients’ clinical information and pathological findings. All parameters of the AP and VP were measured using GSI software based on 70 keV monochrome images. To maintain the accuracy of the measurement results, a round region of interest (ROI) was manually placed in an area with uniform enhancement of the lesion, avoiding areas of calcification, necrosis, and blood vessels that could affect the measurement results. When the lesion was uniform, the area of the ROI should be greater than 1/2 of the largest cross-sectional area of the lesion. When the lesion density was not uniform, the ROI was placed in the area with the most solid components. Meanwhile, to accurately reflect the actual situation of the lesion, the largest cross-section of the lesion and its adjacent layers above and below were selected for measurement, and the average value was calculated. Finally, the average of the two doctors’ measurements was averaged again to obtain the final measurement. In addition, to minimize measurement bias, a copy-and-paste function was used to maintain the position, size, and shape of the ROI consistent in each patient’s two-phase (AP and VP) images. GSI software was used to automatically generate IC, WC, Zeff, CT value at 40 keV (CT_40keV_) and CT value at 70 keV (CT_70keV_) , and calculate the spectral curve slope of the lesion at 70 keV according to the following formula: *λ*_70*keV*_= (*CT*_40 *keV*_–*CT*_70 *keV*_)/70–40)

### Statistical analysis

All statistical analyses were performed using R software (version 3.6.3, https://www.R-project.org ). Continuous and categorical variables are expressed as mean ± standard deviation (SD) and percentage, respectively. Wilcoxon rank sum test was used to compare continuous variables between the SP-TB and S-LUAD groups. Chi-square tests were used to compare categorical variables between two groups. A two-sample *t*-test was used to compare the quantitative parameters of spectral CT between the two groups. Two-sided *P*-value< 0.05 were considered statistically significant. The receiver operating characteristic (ROC) curve was used to evaluate the diagnostic performance of different variables, and the area under the curve (AUC), accuracy, sensitivity, specificity and 95% confidence interval (CI) were calculated.

## Results

### Clinical characteristics of the patients

The clinical characteristics of the patients are summarized in **Table 1**. There were no significant differences in sex, smoking history and lobe location between the SP-TB and S-LUAD groups (*P* = 0.255 for sex, *P* = 0.117 for smoking history, and *P* = 0.100 for lobe location). Age (*P<* 0.001) and carcinoembryonic antigen (CEA) levels (*P<* 0.001) were statistically different between the two groups. Multivariate logistic retrospective analysis showed that age and CEA levels were statistically different between the two groups.

**Table 1:** Clinical characteristics of patients with SP-TB and S-LUAD.

Variable	All Patients (n=246)	SP-TB (n=86)	S-LUAD (n=160)	Univariate analysis	Multivariate analysis	
				*P* value	OR (95% CI)	*P* value
Age (years)				<0.001	1.064 (1.032-1.100)	<0.001
Mean ± SD	53.96 ± 12.45	46.98 ± 12.84	57.71 ± 10.49			
Median (Q_1_, Q_3_)	55.0 (46.8, 63.0)	49.5 (37.0, 56.3)	59.0 (50.0, 64.8)			
Sex (%)				0.255	NA	
Male	141 (57.32%)	54 (62.79%)	87 (54.38%)			
Female	105 (42.68%)	32 (37.21%)	73 (45.63%)			
Smoking history (%)				0.117	NA	
Smoker	72 (29.27%)	31 (36.05%)	41 (25.63%)			
No smoker	174 (70.73%)	55 (63.95%)	119 (74.38%)			
Lobe location (%)				0.100	NA	
RUL	63 (25.61%)	21 (24.42%)	42 (26.25%)			
RML	29 (11.79%)	16 (18.61%)	13 (8.13%)			
RLL	57 (23.17%)	16 (18.61%)	41 (25.63%)			
LIL	54 (21.95%)	21 (24.42%)	33 (20.63%)			
LLL	43 (17.48%)	12 (13.95%)	31 (19.38%)			
CEA (%)				<0.001	19.214 (9.036-45.155)	<0.001
Normal	118 (47.97%)	77 (89.535%)	41 (25.625%)			
High	128 (52.03%)	9 (10.465%)	119 (74.375%)			

CEA, Carcinoembryonic antigen; LLL, Left lower lobe; LUL, Left upper lobe; RLL, Right lower lobe; RML, Right middle lobe; RUL, Right upper lobe; SD, Standard deviation; S-LUAD, Solitary lung adenocarcinoma; SP-TB, Solitary pulmonary tuberculosis.

### Quantitative image analysis

**Figure 1:**
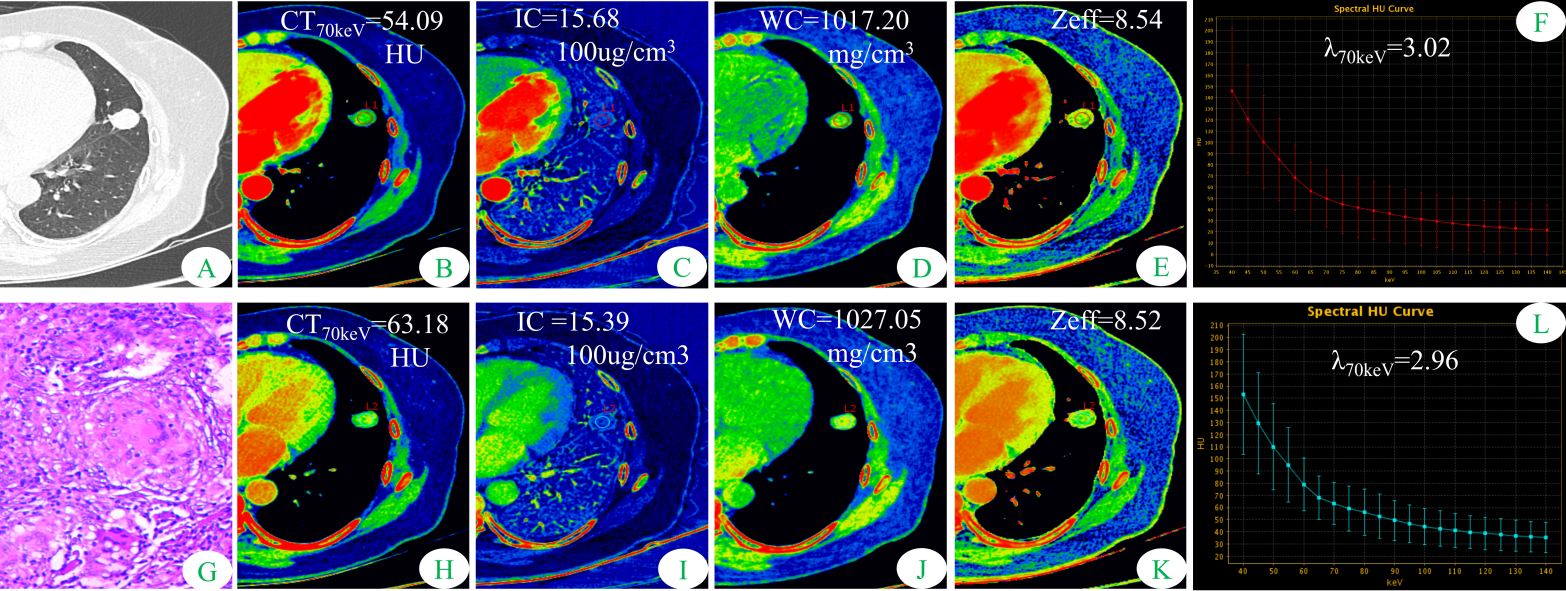
Dual-energy spectral CT images and pathological sections of a 51-year-old woman with solitary pulmonary tuberculosis (SP-TB). **(A)** lung window; **(B, H)** 70keV single-energy pseudo-color images; **(C, I)** iodine concentration (IC) pseudo-color images; **(D, J)** water concentration (WC) pseudo-color images; **(E, K)** effective atomic number (Zeff) pseudo-color images; **(F, L)** spectral curves and slopes; **(G)** hematoxylin and eosin staining (original magnification × 200). **(B–F)** arterial phase; **(H–L)** venous phase.

**Figure 2:**
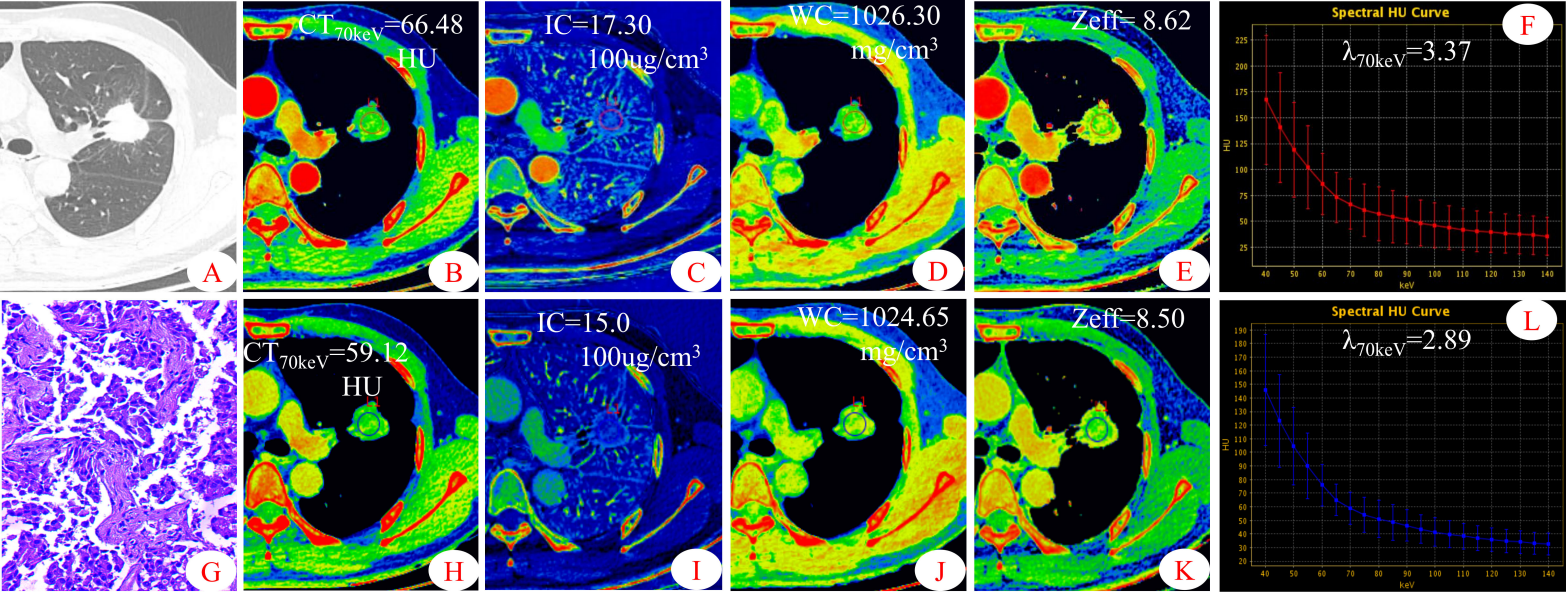
Dual-energy spectral CT images and pathological sections of a 45-year-old man with solitary lung adenocarcinoma (S-LUAD). **(A)** lung window; **(B, H)** 70keV single-energy pseudo-color images; **(C, I)** iodine concentration (IC) pseudo-color images; **(D, J)** water concentration (WC) pseudo-color images; **(E, K)** effective atomic number (Zeff) pseudo-color images; **(F, L)** spectral curves and slopes; **(G)** hematoxylin and eosin staining (original magnification × 200). **(B–F)** arterial phase; **(H–L)**venous phase.

**Figure 3:**
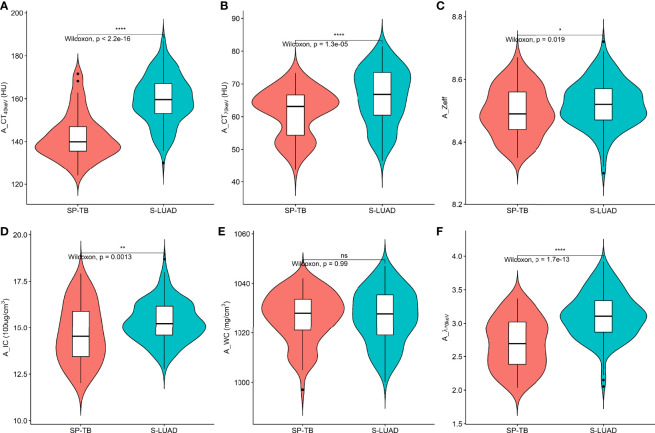
Violin plots of dual-energy spectral CT multiparameter for solitary pulmonary tuberculosis (SP-TB) and solitary lung adenocarcinoma (S-LUAD) in the arterial phase. **(A)** CT_40 keV_ value; **(B)** CT_70 keV_ value; **(C)** effective atomic number (Zeff); **(D)** iodine concentration (IC); **(E)** water concentration (WC); **(F)** spectral curve slope (λ_70 keV_). *P < 0.05; **P < 0.01; ****P < 0.0001; Ns, No significance.

**Figure 4:**
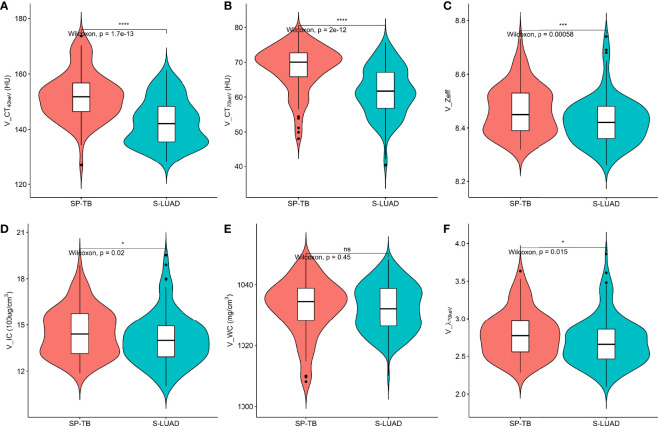
Violin plots of dual-energy spectral CT multiparameter for solitary pulmonary tuberculosis (SP-TB) and solitary lung adenocarcinoma (S-LUAD) in the venous phase. **(A)** CT_40 keV_ value; **(B)** CT_70 keV_ value; **(C)** effective atomic number (Zeff); **(D)** iodine concentration (IC); **(E)** water concentration (WC); **(F)** spectral curve slope (λ_70 keV_). *P < 0.05; ***P < 0.001; ****P < 0.0001; Ns, No significance.

**Figures 1**, **2** show two sets of representative images acquired using spectral CT for patients with SP-TB and S-LUAD, respectively. During the AP, the CT_40keV_ (142.30 ± 10.64 HU vs. 159.75 ± 11.25 HU; P< 0.001), CT_70keV_ (61.35 ± 7.15 HU vs. 66.22 ± 8.50 HU; *P*< 0.001), λ_70keV_ (2.70 ± 0.37 vs. 3.12 ± 0.34; *P*< 0.001), Zeff (8.50 ± 0.08 vs. 8.52 ± 0.07; *P* = 0.015), and IC (14.70 ± 1.57 100ug/cm^3^ vs. 15.32 ± 1.08 100ug/cm^3^; *P* = 0.002) of the SP-TB group were significantly lower than those of the S-LUAD group (all *P*< 0.05) (**Table 2** and **Figure 3**). In the VP, the CT_40keV_ (152.08 ± 8.69 HU vs. 142.49 ± 8.23 HU; *P*< 0.001), CT_70keV_ (68.33 ± 6.57 HU vs. 61.53 ± 6.95 HU; *P*< 0.001), λ_70keV_ (2.79 ± 0.31 vs. 2.70 ± 0.32; *P* = 0.027), Zeff (8.47 ± 0.09 vs. 8.43 ± 0.09; *P* = 0.001), and IC (14.54 ± 1.55 100ug/cm^3^ vs. 14.07 ± 1.65 100ug/cm^3^; *P* = 0.028) of the SP-TB group were significantly higher than those of the S-LUAD group (all *P*< 0.05) (**Table 2** and **Figure 4**). In the AP and VP, WC were not statistically different between the two groups (*P* > 0.05).

**Table 2:** Comparison of spectral CT parameters of SP-TB and S-LUAD in AP and VP.

Parameters	SP-TB (n=86)	S-LUAD (n=160)	*t*	*P*-value
AP_CT_40keV_ (HU)	142.30 ± 10.64	159.75 ± 11.25	-11.82	<0.001
AP_CT_70keV_ (HU)	61.35 ± 8.85	66.22 ± 8.50	-4.76	<0.001
AP_λ_70keV_	2.70 ± 0.37	3.12 ± 0.34	-8.65	<0.001
AP_Zeff	8.50 ± 0.08	8.52 ± 0.07	-2.45	0.015
AP_IC (100ug/cm^3^)	14.70 ± 1.57	15.32 ± 1.08	-3.24	0.002
AP_WC (mg/cm^3^)	1026.52 ± 10.0	1026.64 ± 10.86	-0.99	0.930
VP_CT_40keV_ (HU)	152.08 ± 8.69	142.49 ± 8.23	8.55	<0.001
VP_CT_70keV_ (HU)	68.33 ± 6.57	61.53 ± 6.95	7.46	<0.001
VP_λ_70keV_	2.79 ± 0.31	2.70 ± 0.32	2.22	0.027
VP_Zeff	8.47 ± 0.09	8.43 ± 0.09	3.37	0.001
VP_IC (100ug/cm^3^)	14.54 ± 1.55	14.07 ± 1.65	2.21	0.028
VP_WC (mg/cm^3^)	1032.62 ± 9.03	1032.37 ± 7.64	0.22	0.823

AP, arterial phase; IC, Iodine concentration; S-LUAD, Solitary lung adenocarcinoma; SP-TB, Solitary pulmonary tuberculosis; VP, venous phase; WC, Water concentration; Zeff, Effective atomic number.

### Diagnostic performance of quantitative parameters

**Figure 5:**
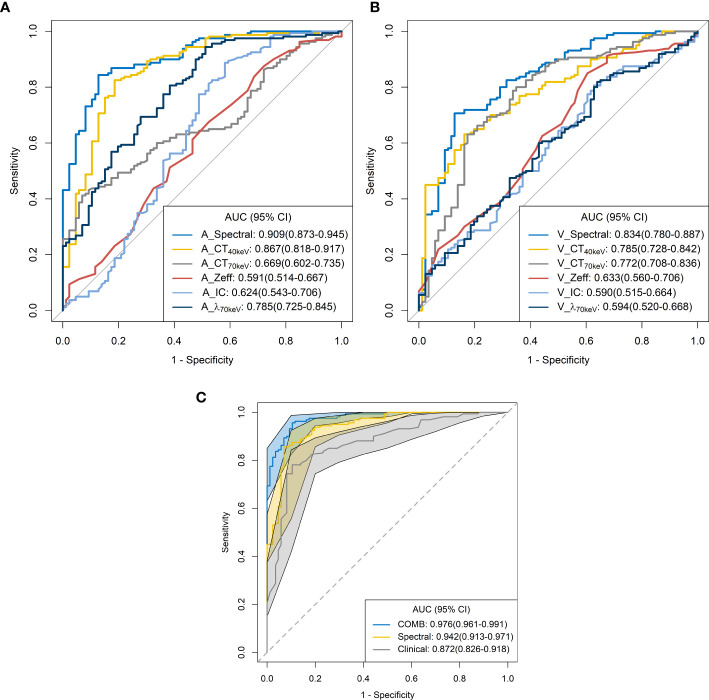
Receiver operating characteristic curves for spectral CT parameters. **(A)** arterial phase; **(B)** venous phase; **(C)** combined.

The ROC curves are presented in **Table 3** and **Figure 5**. Among all single parameters in the AP, the CT_40keV_ value had the highest performance in differentiating SP-TB and S-LUAD, with an AUC, accuracy, sensitivity and specificity of 0.867 (95% CI, 0.818 – 0.917), 0.821 (95% CI, 0.767 – 0.867), 0.825 (95% CI, 0.656 – 0.887) and 0.814 (95% CI, 0.663 – 0.884), respectively. When all the single parameters of the AP were combined, the diagnostic performance was further improved, with AUC, accuracy, sensitivity and specificity of 0.867 (95% CI, 0.818 – 0.917), 0.821 (95% CI, 0.767 – 0.867), 0.825, respectively (95% CI, 0.656 – 0.887) and 0.814 (95% CI, 0.663 – 0.884), respectively (**Table 3**; **Figure 5A**). Similarly, when all the single parameters in the VP were combined, the diagnostic performance was higher than that of the single parameters, with an AUC, accuracy, sensitivity and specificity of 0.834 (95% CI, 0.780 – 0.887), 0.764 (95% CI, 0.706 – 0.816), 0.706 (95% CI, 0.419 – 0.769), and 0.872 (95% CI, 0.662 – 0.919), respectively (**Table 3**; **Figure 5C**).

**Table 3:** ROC curve analysis of spectral CT parameters in distinguishing between SP-TB and S-LUAD.

Parameters	AUC (95% CI)	Cutoff	Accuracy (95% CI)	Sensitivity (95% CI)	Specificity (95% CI)
AP					
CT_40keV_ (HU)	0.867(0.818-0.917)	149.52	0.821(0.767-0.867)	0.825(0.656-0.887)	0.814(0.663-0.884)
CT_70keV_ (HU)	0.669(0.602-0.735)	69.66	0.593(0.529-0.655)	0.412(0.256-0.506)	0.930(0.755-0.965)
λ_70keV_	0.785(0.725-0.845)	2.65	0.785(0.728-0.834)	0.944(0.800-0.981)	0.488(0.349-0.605)
Zeff	0.591(0.514-0.667)	8.45	0.667(0.604-0.725)	0.875(0.731-0.946)	0.279(0.163-0.384)
IC (100ug/cm^3^)	0.624(0.543-0.706)	14.08	0.724(0.663-0.778)	0.887(0.706-0.938)	0.419(0.256-0.523)
AP_all	0.909(0.873-0.945)	0.61	0.854(0.803-0.895)	0.844(0.669-0.894)	0.872(0.686-0.930)
VP					
CT_40keV_ (HU)	0.785(0.728-0.842)	144.91	0.703(0.642-0.760)	0.631(0.481-0.738)	0.826(0.698-0.896)
CT_70keV_ (HU)	0.772(0.708-0.836)	63.23	0.699(0.638-0.756)	0.631(0.331-0.731)	0.826(0.698-0.896)
λ_70keV_	0.594(0.520-0.668)	2.92	0.659(0.596-0.718)	0.819(0.594-0.887)	0.360(0.163-0.454)
Zeff	0.633(0.560-0.706)	8.50	0.691(0.629-0.748)	0.850(0.660-0.931)	0.395(0.276-0.498)
IC (100ug/cm^3^)	0.590(0.515-0.664)	15.11	0.654(0.591-0.714)	0.806(0.637-0.875)	0.372(0.244-0.477)
VP_all	0.834(0.780-0.887)	0.69	0.764(0.706-0.816)	0.706(0.419-0.769)	0.872(0.662-0.919)
Clinical	0.872(0.826-0.918)	0.65	0.821(0.767-0.867)	0.781(0.531-0.856)	0.895(0.744-0.954)
AP_all + VP_all	0.942(0.913-0.971)	0.70	0.882(0.835-0.920)	0.856(0.500-0.913)	0.930(0.814-0.965)
Combined*	0.976(0.961-0.991)	0.54	0.935(0.897-0.962)	0.956(0.812-0.988)	0.895(0.732-0.953)

AP, arterial phase; AUC, Area under the curve; CI, Confidence interval; IC, Iodine concentration; ROC, Receiver operating characteristic;

S-LUAD, Solitary lung adenocarcinoma; SP-TB, Solitary pulmonary tuberculosis; VP, venous phase; Zeff, Effective atomic number.

* Combined: AP_all + VP_all + ClinicalFigure legends.

When clinical variables (AUC, 0.872; 95% CI, 0.826 – 0.918) were combined with all parameters of spectral CT (AUC, 0.942; 95% CI, 0.913 – 0.971), the diagnostic performance was highest, and the AUC, accuracy, sensitivity and specificity were 0.976 (95% CI, 0.961-0.991), 0.935 (95% CI, 0.897 – 0.962), 0.956 (95% CI, 0.812 – 0.988), and 0.895 (95% CI, 0.732 – 0.953), respectively (**Table 3**; **Figure 5C**).

## Discussion

In this study, we used dual-energy spectral CT to distinguish solitary pulmonary tuberculosis from solitary lung adenocarcinoma. Spectral CT parameters combined with clinical variables showed excellent predictive performance (AUC, 0.976; 95% CI, 0.961 – 0.991) in identifying these two diseases.

According to previous reports (17, 18), lung cancer patients were older than tuberculosis patients, and the expression rate of CEA in lung cancer patients was higher. This is consistent with our findings. Pulmonary tuberculosis tends to involve the posterior apical segment of the upper lobe and the dorsal segment of the lower lobe of both lungs. However, this trend was not observed in this study. This may be related to our smaller sample size, or to the type of tuberculosis, which needs further study.

Some parameters of spectral CT can indirectly reflect the intrinsic characteristics of the tumor, such as CT value and IC, which can indirectly reflect the blood supply to the tumor, and Zeff can reflect the effective atomic number of inorganic substances in the tumor (4, 19, 20). Therefore, these parameters can be used as imaging markers for lung tumors. The results of this study showed that in the AP, the CT_40keV_ value, CT_70keV_ value, λ_70keV_, IC, and Zeff of the S-LUAD group were significantly higher than those of the SP-TB group, whereas during the VP, these spectral CT parameters were significantly increased in the SP-TB group. LUAD usually causes new blood vessels and an abundant vascular network (21), and TB has a relatively insufficient microvessel density. These changes may have contributed to the differences in spectral CT parameters between the two groups. Iodine is the main component of CT contrast agents, and IC in the tumor reflects its degree of enhancement, which can indirectly reflect the relative vascular distribution in the tumor (22). The energy spectral curve describes the attenuation of different tissues under X-ray irradiation at various energies. Therefore, different tissues have characteristic energy spectral curves (23). The difference in the energy spectral curve was evaluated by calculating the slope of the curve. In this study, CT values at low energies (40 keV and 70 keV) were selected, and the slope of the curve at 70 keV was calculated. The results showed that in the AP, the energy spectral curve of the S-LUAD group was relatively steep and the curve slope was larger, and the energy spectral curve of the SP-TB group was relatively flat and the curve slope was smaller, whereas in the VP, the results were opposite (**Figures 1**, **2**). However, this was inconsistent with the findings of Hou et al. (24). This may be due to the fact that they only explored the relationship between inflammatory masses and lung cancer, and did not make a detailed distinction between the pathological types of these lesions. Therefore, our study may be more in line with the clinical practice.

ROC curve analysis showed that the combined diagnostic performance of all spectral parameters in the AP and VP was higher than that of other single parameters (**Table 3**; **Figures 5A,B**). All spectral parameters combined with clinical variables showed the highest diagnostic performance (AUC = 97.6%) in distinguishing SP-TB and S-LUAD (**Table 3**; **Figure 5C**). However, the population assessed in this study was based on a small geographic area, and the stability of the results needs to be further verified in larger sample sizes and more centers. These findings suggest that spectral CT has potential clinical value for differentiating between these two diseases.

Our study had several limitations. First, similar to other retrospective studies, this study may have an inherent selection bias. In future studies, more patients should be recruited from multiple centers to limit bias as much as possible. Second, this study only discussed the predictive performance of spectral CT in distinguishing SP-TB from S-LUAD; therefore, the predictive performance of spectral CT for other lung cancer subtypes should be further investigated in the future to draw broader conclusions. Third, more pathological information may provide experimental pathophysiological rationale for the findings, however, this study lacks the correlation between spectral CT parameters and pathological information.

In conclusion, dual-energy spectral CT is a promising method to distinguish SP-TB from S-LUAD. This preliminary result should be further validated in a multicenter and large sample, so that this non-invasive and effective technique can be applied in clinical practice.

## Data availability statement

The original contributions presented in the study are included in the article/supplementary material. Further inquiries can be directed to the corresponding authors.

## Ethics statement

The studies involving human participants was reviewed and approved by the medical ethics committees of Lanzhou University Second Hospital. The ethics committee waived the requirement to participate in written informed consent.

## Author contributions

GZ, ShL, KY, and WK contributed to conception and design of the study. GZ, ShL, SL, and FZ organized the database. GZ, JR, and QM performed the statistical analysis. GZ, ShL, KY, SL, FZ, ZH, QM, and WK wrote the first draft of the manuscript. JR, ZZ, JZ, HP, and QM wrote sections of the manuscript. All authors contributed to manuscript revision, read, and approved the submitted version.

## Funding

This work was supported by the National Natural Science Foundation of China (No. 82202147), the Sichuan Provincial Department of Science and Technology Project (No. 2022YFS0075), the Sichuan Provincial Cadre Health Research Project (No. 2022-208, 2020-225, and 2021-230), and the Sichuan Academy of Medical Sciences & Sichuan Provincial People’s Hospital Research Fund (No. 2022QN25).

## Conflict of interest

Author JR was employed by GE Healthcare.

The remaining authors declare that the research was conducted in the absence of any commercial or financial relationships that could be construed as a potential conflict of interest.

## Publisher’s note

All claims expressed in this article are solely those of the authors and do not necessarily represent those of their affiliated organizations, or those of the publisher, the editors and the reviewers. Any product that may be evaluated in this article, or claim that may be made by its manufacturer, is not guaranteed or endorsed by the publisher.

## References

[1] Sung H Ferlay J Siegel RL Laversanne M Soerjomataram I Jemal A. Global cancer statistics 2020: GLOBOCAN estimates of incidence and mortality worldwide for 36 cancers in 185 countries. CA: Cancer J Clin (2021) 71(3):209–49. doi: 10.3322/caac.21660 33538338

[2] Ettinger DS Wood DE Aisner DL Akerley W Bauman JR Bharat A. NCCN guidelines insights: Non-small cell lung cancer, version 2.2021. J Natl Compr Cancer Network JNCCN (2021) 19(3):254–66. doi: 10.6004/jnccn.2021.0013 33668021

[3] Harris K Puchalski J Sterman D. Recent advances in bronchoscopic treatment of peripheral lung cancers. Chest (2017) 151(3):674–85. doi: 10.1016/j.chest.2016.05.025 27292045

[4] Zhang G Cao Y Zhang J Zhao Z Zhang W Huang L. Focal organizing pneumonia in patients: differentiation from solitary bronchioloalveolar carcinoma using dual-energy spectral computed tomography. Am J Transl Res (2020) 12(7):3974–83. PMC7407697 32774750

[5] Wang XL Shan W. Application of dynamic CT to identify lung cancer, pulmonary tuberculosis, and pulmonary inflammatory pseudotumor. Eur Rev Med Pharmacol Sci (2017) 21(21):4804–09. Available at: https://www.europeanreview.org/article/13720. 29164583

[6] Qi L-P Chen K-N Zhou XJ Tang L Liu Y-L Li X-T. Conventional MRI to detect the differences between mass-like tuberculosis and lung cancer. J Thorac Dis (2018) 10(10):5673–84. doi: 10.21037/jtd.2018.09.125 PMC6236172 30505475

[7] Lim CG Shin KM Lim JS Lim JK Kim HJ Kim WH. Predictors of conversion to thoracotomy during video-assisted thoracoscopic surgery lobectomy in lung cancer: additional predictive value of FDG-PET/CT in a tuberculosis endemic region. J Thorac Dis (2017) 9(8):2427–36. doi: 10.21037/jtd.2017.07.40 PMC5594172 28932548

[8] Drumm O Joyce EA de Blacam C Gleeson T Kavanagh J McCarthy E. CT-guided lung biopsy: Effect of biopsy-side down position on pneumothorax and chest tube placement. Radiology (2019) 292(1):190–96. doi: 10.1148/radiol.2019182321 31084480

[9] Tai R Dunne RM Trotman-Dickenson B Jacobson FL Madan R Kumamaru KK. Frequency and severity of pulmonary hemorrhage in patients undergoing percutaneous CT-guided transthoracic lung biopsy: Single-institution experience of 1175 cases. Radiology (2016) 279(1):287–96. doi: 10.1148/radiol.2015150381 26479161

[10] Dubé J-P Azzi Z Semionov A Sayegh K Kosiuk J Pressacco J. Imaging of post transthoracic needle biopsy complications. Can Assoc Radiol J (2019) 70(2):156–63. doi: 10.1016/j.carj.2018.08.006 30635216

[11] Hu Y Zhao X Zhang J Han J Dai M. Value of f-FDG PET/CT radiomic features to distinguish solitary lung adenocarcinoma from tuberculosis. Eur J Nucl Med Mol Imaging (2021) 48(1):231–40. doi: 10.1007/s00259-020-04924-6 32588088

[12] Yang X Dong X Wang J Li W Gu Z Gao D. Computed tomography-based radiomics signature: A potential indicator of epidermal growth factor receptor mutation in pulmonary adenocarcinoma appearing as a subsolid nodule. oncologist (2019) 24(11):e1156–e64. doi: 10.1634/theoncologist.2018-0706 PMC6853103 30936378

[13] Zhang G Cao Y Zhang J Zhao Z Zhang W Zhou J. Epidermal growth factor receptor mutations in lung adenocarcinoma: associations between dual-energy spectral CT measurements and histologic results. J Cancer Res Clin Oncol (2021) 147(4):1169–78. doi: 10.1007/s00432-020-03402-8 PMC11802015 32980961

[14] Jia Y Xiao X Sun Q Jiang H. CT spectral parameters and serum tumour markers to differentiate histological types of cancer histology. Clin Radiol (2018) 73(12):1033–40. doi: 10.1016/j.crad.2018.07.104 30115364

[15] Lin LY Zhang Y Suo ST Zhang F Cheng JJ Wu HW. Correlation between dual-energy spectral CT imaging parameters and pathological grades of non-small cell lung cancer. Clin Radiol (2018) 73(4):412. doi: 10.1016/j.crad.2017.11.004 29221718

[16] Hong SR Hur J Moon YW Han K Chang S Kim JY. Predictive factors for treatment response using dual-energy computed tomography in patients with advanced lung adenocarcinoma. Eur J Radiol (2018) 101:118–23. doi: 10.1016/j.ejrad.2018.02.019 29571784

[17] Leng S Zheng J Jin Y Zhang H Zhu Y Wu J. Plasma cell-free DNA level and its integrity as biomarkers to distinguish non-small cell lung cancer from tuberculosis. Clin Chim Acta (2018) 477:160–65. doi: 10.1016/j.cca.2017.11.003 29113814

[18] Parker CS Siracuse CG Litle VR. Identifying lung cancer in patients with active pulmonary tuberculosis. J Thorac Dis (2018) 10(Suppl 28):S3392–S97. doi: 10.21037/jtd.2018.07.11 PMC6218365 30505526

[19] Goo HW Goo JM. Dual-energy CT: New horizon in medical imaging. Korean J Radiol (2017) 18(4):555–69. doi: 10.3348/kjr.2017.18.4.555 PMC5447632 28670151

[20] Hamid S Nasir MU So A Andrews G Nicolaou S Qamar SR. Clinical applications of dual-energy CT. Korean J Radiol (2021) 22(6):970–82. doi: 10.3348/kjr.2020.0996 PMC8154785 33856133

[21] Yazdani S Miki Y Tamaki K Ono K Iwabuchi E Abe K. Proliferation and maturation of intratumoral blood vessels in non-small cell lung cancer. Hum Pathol (2013) 44(8):1586–96. doi: 10.1016/j.humpath.2013.01.004 23522064

[22] Knöss N Hoffmann B Krauss B Heller M Biederer J. Dual energy computed tomography of lung nodules: differentiation of iodine and calcium in artificial pulmonary nodules in vitro. Eur J Radiol (2011) 80(3):e516-e19. doi: 10.1016/j.ejrad.2010.11.001 21112712

[23] Deniffel D Sauter A Dangelmaier J Fingerle A Rummeny EJ Pfeiffer D. Differentiating intrapulmonary metastases from different primary tumors *via* quantitative dual-energy CT based iodine concentration and conventional CT attenuation. Eur J Radiol (2019) 111:6–13. doi: 10.1016/j.ejrad.2018.12.015 30691666

[24] Hou WS Wu HW Yin Y Cheng JJ Zhang Q Xu JR. Differentiation of lung cancers from inflammatory masses with dual-energy spectral CT imaging. Acad Radiol (2015) 22(3):337–44. doi: 10.1016/j.acra.2014.10.004 25491737

